# Modulation of Cosmogenic Tritium in Meteoric Precipitation by the 11-year Cycle of Solar Magnetic Field Activity

**DOI:** 10.1038/s41598-018-31208-9

**Published:** 2018-08-24

**Authors:** László Palcsu, Uwe Morgenstern, Jürgen Sültenfuss, Gabriella Koltai, Elemér László, Marjan Temovski, Zoltán Major, Judit T. Nagy, László Papp, Carmen Varlam, Ionut Faurescu, Marianna Túri, László Rinyu, György Czuppon, Emese Bottyán, A. J. Timothy Jull

**Affiliations:** 1 Isotope Climatology and Environmental Research Centre (ICER), Institute for Nuclear Research, Hungarian Academy of Sciences, Debrecen, 4026 Hungary; 2 GNS Science, Lower Hutt, 5040 New Zealand; 3 Institute of Environmental Physics, University of Bremen, Bremen, 28359 Germany; 4 Institute of Geology, University of Innsbruck, Innsbruck, 6020 Austria; 5 Doctoral School of Mathematical and Computational Sciences, University of Debrecen, Debrecen, 4032 Hungary; 6 National R&D Institute of Cryogenics and Isotopic Technologies, Râmnicu Vâlcea, 240050 Romania; 7 Institute for Geological and Geochemical Research, Research Centre for Astronomy and Earth Sciences, Hungarian Academy of Sciences, Budapest, 1112 Hungary; 8 Department of Geosciences, University of Arizona, Tucson, AZ 85721 USA

## Abstract

The relationship between the atmospheric concentration of cosmogenic isotopes, the change of solar activity and hence secondary neutron flux has already been proven. The temporal atmospheric variation of the most studied cosmogenic isotopes shows a significant anti-correlation with solar cycles. However, since artificial tritium input to the atmosphere due to nuclear-weapon tests masked the expected variations of tritium production rate by three orders of magnitude, the natural variation of tritium in meteoric precipitation has not previously been detected. For the first time, we provide clear evidence of the positive correlation between the tritium concentration of meteoric precipitation and neutron flux modulated by solar magnetic activity. We found trends in tritium time series for numerous locations worldwide which are similar to the variation of secondary neutron flux and sun spot numbers. This variability appears to have similar periodicities to that of solar cycle. Frequency analysis, cross correlation analysis, continuous and cross wavelet analysis provide mathematical evidence that the correlation between solar cycle and meteoric tritium does exist. Our results demonstrate that the response of tritium variation in precipitation to the solar cycle can be used to help us understand its role in the water cycle.

## Introduction

Cosmic rays are composed of high-energy charged particles, mainly originating from outside the solar system (galactic cosmic rays, GCR), although there are also lower-energy solar cosmic rays (SCR). When cosmic rays reach the Earth’s atmosphere, a cascade of secondary particles and nuclei are produced in numerous nuclear reactions between these secondary particles and the atmospheric nuclei. Isotopes formed in such reactions are called cosmogenic isotopes^1–3^. The secondary neutrons (a part of the hadronic component) produce – for example – the well-known cosmogenic isotopes: ^14^C and ^3^H are produced mainly in ^14^N(n,p)^14^C and ^14^N(n,^3^H)^12^C reactions, while ^10^Be and ^7^Be are formed mainly by spallation of oxygen and nitrogen^4–6^. Hence, the temporal and spatial variations of cosmogenic isotopes in the environment can be used as tracers of different atmospheric, hydrological or geochemical processes^7^.

Tritium production is different from ^14^C: the latter is caused by capture of a thermal neutron, while the former is a threshold-like reaction requiring at least ~4 MeV energy of the neutron. Long-term changes are generally attributed to geomagnetic field effects, but short-term changes in cosmogenic isotope production rates are primarily driven by the magnetic field variation of the Sun. The Earth’s geomagnetic field modulated by the solar wind allows charged cosmogenic particles to enter the atmosphere at different threshold energies from the Equator to the poles. The cutoff rigiditiy varies from 17 GV (Asian Equator region) to 1 GV (polar regions)^8^. As a result, the cosmogenic production ratio is higher towards the poles.

The solar cycle is commonly thought to be the 11-year cycle of the sun spot numbers (Schwabe cycle). In reality, sunspots are magnetic flux tube emergences at the Sun’s surface. A solar cycle covers a 22-year magnetic pole change (Hale-cycle), during which the strength of the Sun’s magnetic field alternates with a period of ~11 years. The strength of the GCR radiation at the Earth depends on the magnetic field of the Sun: the stronger the solar magnetic field, the lower the flux of the cosmic rays reaching the inner Solar System. Besides GCR, energetic particles come also from the Sun. Theoretical calculations have shown that the solar wind is of minor importance or negligible regarding the production rate of cosmogenic isotopes^9^. As for ^14^C, the mean contribution of solar energetic particles for the last 50 years is estimated to be 0.25%, although SCR events can cause short-term excursions of up to ~2.2% in ^14^C, as recorded in tree rings^10–12^, but these excursions are rare, non-periodic, and have only been detected for a few specific time periods.

**Figure 1:**
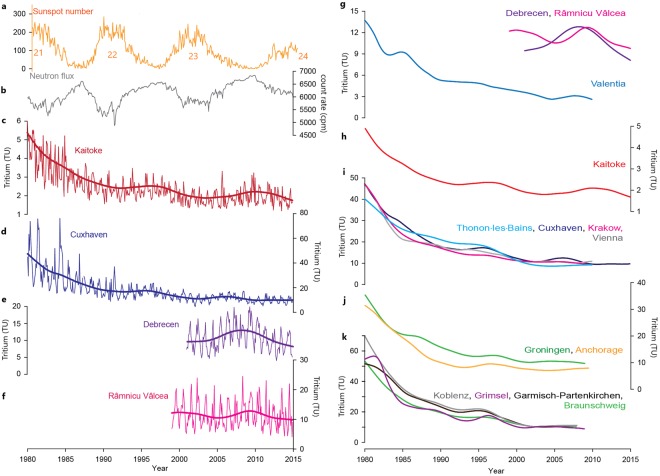
Time series of selected parameters. (**a**) Sunspot numbers in time, the number of the solar cycle is also indicated; (**b**) neutron count rate (Oulu, Finland); (**c**–**f**) tritium time series with spline average trends in four selected location (Kaitoke, New Zealand; Cuxhaven, Germany; Debrecen, Hungary; Râmnicu Vâlcea, Romania); (**g**–**k**) tritium spline trends in fourteen locations.

Neutron monitor (NM) data are widely compared to production rates of cosmogenic isotopes in the atmosphere. Neutron monitor stations over the world show that the variation of secondary neutrons (detected in monitor stations in a modulated manner) follows the solar cycle^13–15^. NM count rates are related to the flux of secondary nucleons near ground and are not equal, however, strongly related to the neutron flux. The amplitude of the NM-recorded 11-year cycle depends on location and ranges from a few % to 15% between equatorial and polar regions^15^. The neutron monitor data of a specific station depend on the modelling used for the particular monitor as well as the fact they are at ground level. The magnitude of the variation in Oulu (Finland) is 16–20% compared to the average neutron count rate in a solar cycle, as can be seen in Fig. 1b. The production rate of cosmogenic isotopes depends mainly on the secondary neutron flux, hence the change in the production rate that follows the change in the neutron radiation.

The temporal correlation between the number of cosmogenic nuclei and the solar cycle has already been shown, however, a direct relation for tritium has not been demonstrated so far. Nevertheless, neutron flux variations alone cannot provide an explanation for the temporal changes of cosmogenic isotope concentrations in different archives. Other factors, related to transport and reservoirs, seem to have an influence on the amplitude and delay of the temporal variation of their concentration caused by the solar cycle^16^. For instance, the ^14^C-content of tree rings shows only a ±2–3‰ change during the 11-year cycle^17^. This weak variability in case of atmospheric ^14^C can be explained by the large CO_2_ reservoir in the atmosphere with a long residence time of about 50–100 years that dilutes any variability from short-term effects^18,19^.

In contrast, ^7^Be and ^10^Be variabilities are characterised by a slightly larger amplitude than what can be attributed to changes in the production ratio^20^. During a solar cycle, tropospheric ^7^Be concentrations in atmospheric aerosols vary by about ±15%, which indeed exceeds the change in neutron count rate variability. After ^7^Be has been produced in the upper troposphere or the lower stratosphere, it is immediately adsorbed to aerosols, and reaches the surface as dry and/or wet fall-out. As this process takes only a few weeks in the troposphere, the ^7^Be formed appears on the Earth’s surface shortly after its production^21^. Moreover, the atmosphere does not have a reservoir for beryllium isotopes that could dilute the variation of the production rate. Although, stratospheric beryllium has a residences time of 1–2 years^15^, it affects its temporal variation of the concentration in precipitation of only ^10^Be (half-life: 1.4 million years), since ^7^Be is vanishing faster due to its short half-life (53 days).

In nature, tritium (^3^H) is also produced due to secondary neutrons^22^. ^3^H oxidizes rapidly to H^3^HO, and enters the global water cycle. Earlier theoretical studies have estimated a tritium production rate of 0.25 atoms cm^−2^ s^−1^^3,22^. It is hard to observe this production intensity directly. A few aircraft missions have attempted to examine hydrogen isotope variations including ^3^H in upper tropospheric and lower stratospheric water vapour^23^. However, the temporal resolution and duration of such missions are not suitable to investigate long term changes of tritium production rate. Instead, the tritium concentration of meteoric precipitation (rain, snow) is worth analysing even though it is influenced by a combination of processes apart from its production rate. These mechanisms include, among other atmospheric processes, troposphere/stratosphere interactions, cloud formation and re-evaporation of surface waters^24,25^.

In addition, in the 1960’s due to atmospheric thermonuclear weapon tests, the tritium concentration of precipitation in both hemispheres was increased by 2‒3 orders of magnitude, obscuring natural variations^26–28^. In the summer of 1963, the tritium in precipitation increased to 6000 TU (Supplementary Fig. 1) in temperate continental regions in the northern hemisphere (1 TU represents a ^3^H/^1^H ratio of 1 × 10^−18^.). This large tritium peak is called the ‘bomb-peak’, and this artificial tritium is referred as ‘bomb tritium’. After the Nuclear Test Ban Treaty was ratified by the USA and the Soviet Union in 1963, atmospheric tritium levels started decreasing as a result of its radioactive decay and atmospheric removal by precipitation. The attenuation of the tritium bomb peak was still present in the 80’s as well as in the 90’s (Supplementary Fig. 1).

However, since the late 90’s this decreasing trends is no longer present, implying that tritium in precipitation may have reached a steady state level. The Kaitoke record, with absence of artificial and recycled bomb tritium in this oceanic environment, strongly supports this interpretation. However, this value still exceeds the natural level in Europe and North America, which is assumed to be 5–8 TU for the European temperate regions^29^. In Debrecen, Hungary, the average between 2001 and 2014 was 11.2 TU (Fig. 1g). This 3–6 TU excess can be attributed to global emissions from nuclear facilities^30^. Before the nuclear epoch, very low levels of natural tritium could not be analysed with appropriate accuracy. Since the late 70’s, precise measurements can be performed by proportional and liquid scintillation counting techniques with electrolytic enrichment and mass spectrometrically using the ^3^He-ingrowth method^31,32^. Although numerous tritium time series of meteoric water exist worldwide, clear evidence of a direct link between the solar cycle and the tritium concentration of precipitation has not been presented up to now.

## Results

### Evaluation of tritium time series of precipitation

In this study, we examine the long-term changes of tritium content of precipitation in order to investigate how the solar cycle influences the tritium amounts. We have been taking event-based and monthly precipitation samples for tritium analysis in Râmnicu Vâlcea (Romania) and in Debrecen (Hungary) since 1999 and 2001, respectively. In addition, tritium data series taken from Cuxhaven, Germany and Kaitoke, New Zealand and twelve other locations from the GNIP database (IAEA/WMO 2017)^26^ were also studied. The analytical accuracy of our dataset (see Methods) makes it possible to detect predicted natural tritium variations^33–36^. Altogether 16 complete tritium time series, sun spot numbers, ^7^Be and ^10^Be time series have been analysed, compared and correlated to neutron count rate values (all time series are available in the Supplementary Information).

**Figure 2:**
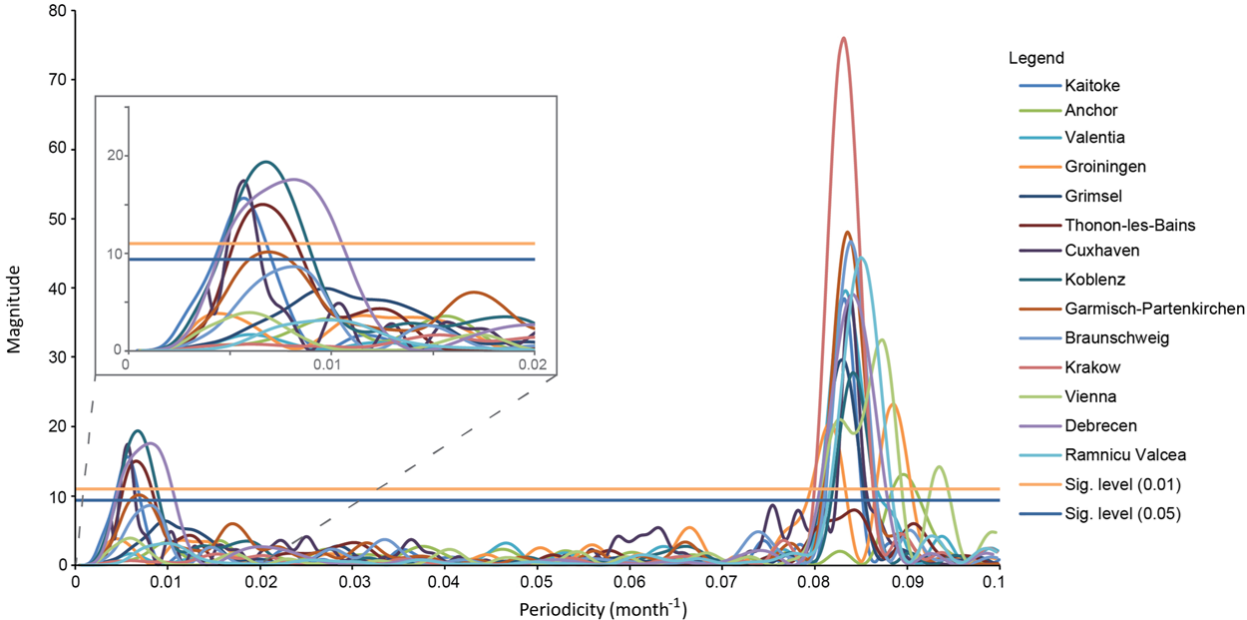
Frequency (periodicity) analysis of the tritium data from 1990 to 2014. The frequency distribution taking data from 1990 to 2014 shows a significant periodicity at around 0.083 month^−1^ (12 month), representing the well-known one-year seasonal frequency of the tritium time series, while the second significant frequency results in a period of 12.4 ± 1.8 years.

Figure 1 shows tritium time series of different stations from 1980, secondary neutron count rate, in Oulu, Finland and sunspot numbers. The decreasing trend in tritium time series in the 80′s can be attributed to the attenuating contribution of the bomb tritium, while in the last two decades the observed tritium concentration of precipitation has decreased close to the natural level. When the tritium reached its steady-state level in the early 1990′s (from the middle of solar cycle 22) a local increase trend started evolving in the tritium time series for most of the locations. A similar trend starting from 2002 (the middle of solar cycle 23) is present at many stations, including Kaitoke, Cuxhaven, Koblenz, Garmisch-Partenkirchen, Braunschweig, Grimsel, Debrecen, Râmnicu Vâlcea. The highest tritium concentrations show up at the end of the solar cycle 23. These local maxima appear to coincide with neutron count rate maxima. To prove the potential correlation between tritium in precipitation and neutron count rate and hence solar cycle, several mathematical analyses have been carried out (frequency analysis with fast Fourier transform algorithm, cross correlation analysis, continuous and wavelet coherence analyses^37^). Figure 2 shows the frequency distribution obtained for all tritium time series taking data from 1990 to 2014, exhibiting two significant frequency ranges. The first one is centered around 0.083 month^−1^, representing the well-known one-year seasonal frequency of the tritium time series, while the second significant frequency is (6.74 ± 0.95) × 10^−3^ month^−1^, which results in a period of 12.4 ± 1.8 years. When the frequency analysis is performed for the whole 25-year time span, from 1980 to 2014, an additional frequency appears at around 2.5 × 10^−3^ month^−1^ (28 years) (Supplementary Fig. 2). This apparent periodicity appears to be due to the attenuation of the bomb contribution, which was still substantial in the 80’s. Excluding data from the 80’s, this frequency does not come up. From 1990, the tritium of the precipitation can be considered as free of the contribution of the nuclear weapon tests taken place in the 50’s and 60’s. The average duration of the historically described 23 solar cycles is 11.0 ± 1.8 years, while that of the last three cycles (from 1976 to 2008) is 10.6 ± 1.8 years. We obtained 12.4 ± 1.8 years as the periodicity of tritium variation, which is in a good agreement with the 11-year solar cycle.

To further confirm the correlation between the solar cycle and tritium of precipitation, cross-correlation analyses and wavelet transform analyses were performed. Spline average and bomb peak corrected time series of tritium concentration of precipitation from fourteen stations were applied for cross-correlation analysis. These cross-correlation calculations further corroborate the positive correlation between neutron count rate and tritium time series at most of the locations (Supplementary Fig. 3). Cross-correlation coefficients are weaker in the case of spline-averaged tritium time series (without bomb correction), which can be explained by the attenuating contribution of artificial tritium from nuclear weapon experiments. Stronger cross-correlation coefficients (at 95% of confidence level) were found in the case of the bomb peak corrected time series, where the bomb contribution had been subtracted from the time series.

**Figure 3:**
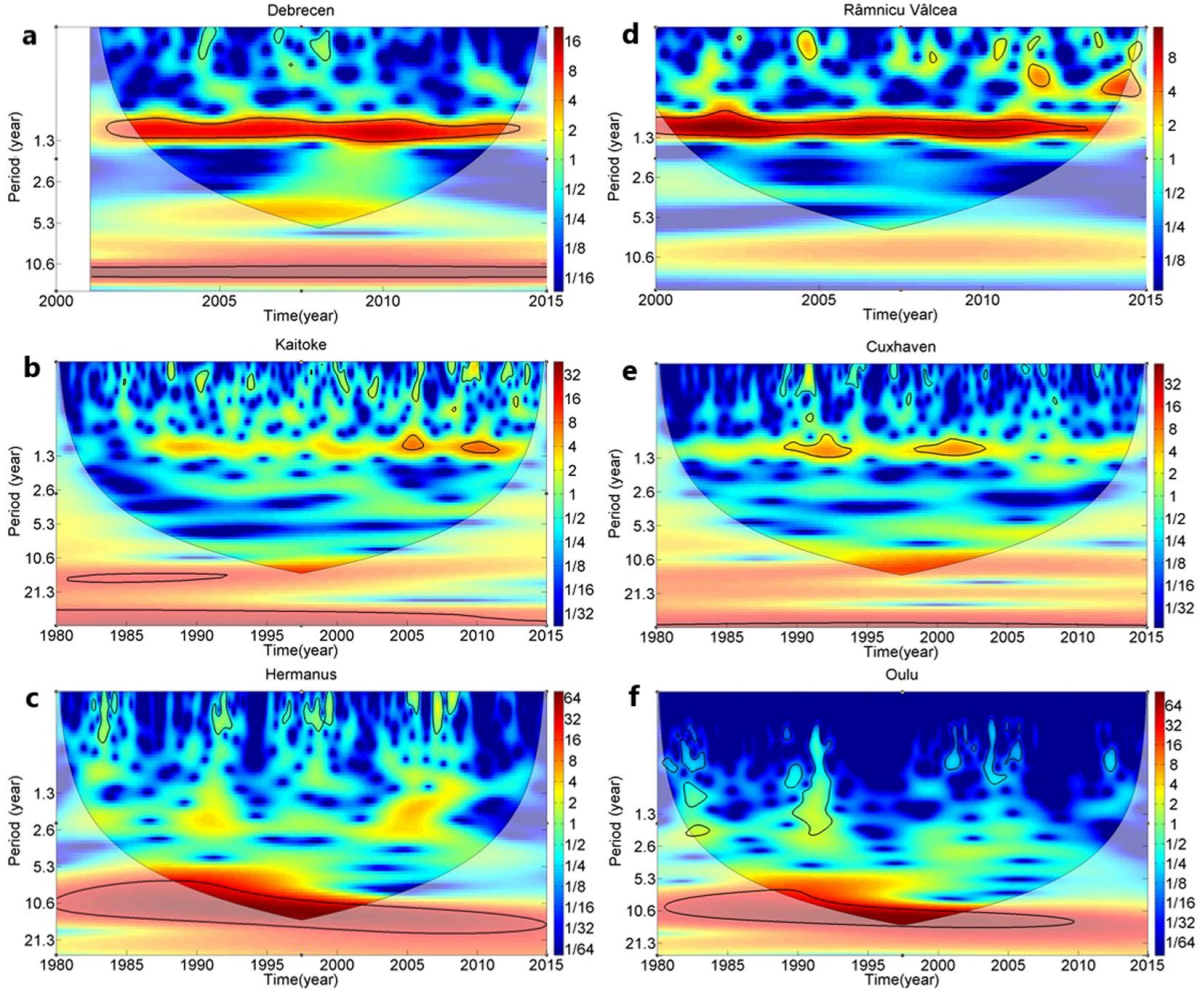
Wavelet analysis. Continuous Wavelet analyses for tritium time series for four locations (**a**) Debrecen, (**d**) Râmnicu Vâlcea, (**b**) Kaitoke, (**e**) Cuxhaven) and neutron flux monitors at (**c**) Oulu (Finland) and (**f**) Hermanus (South Africa).

**Figure 4:**
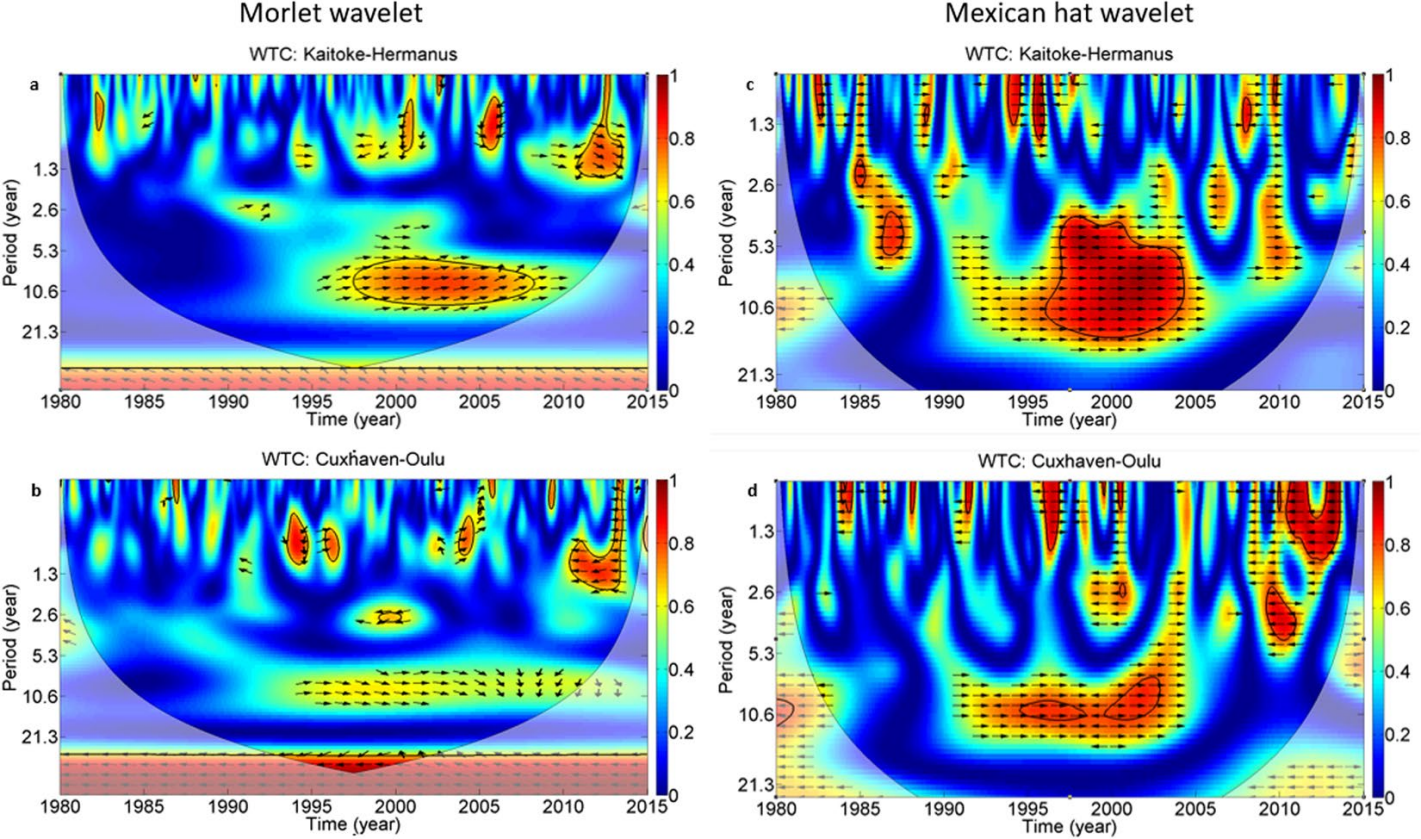
Wavelet coherence analyses for Cuxhaven and Kaitoke between tritium concentration of precipitation and neutron count rate of Oulu (for Cuxhaven) and Hermanus (for Kaitoke). Red areas bounded by black contour lines indicate significant coherence (α < 0.05). The shading indicates areas outside the cone of influence. Arrows indicate phase angle (in the all case of time series are in phase when the arrows point to the right).

Wavelet transform analyses exploit the spectral power of frequency and cross-correlation patterns in time. Continuous wavelet calculations also show a 12-month period in all cases (Fig. 3). This is in a good agreement with the well known seasonality of tritium concentration of precipitation^26–30^. Furthermore, a significant period appears at around ~11–13 years in the middle of the investigated time interval. This 11-year period is obviously seen in the neutron count rate time series, while for Kaitoke and Cuxhaven the 11-year period is weaker but still significant. The data from Debrecen and Râmnicu Vâlcea constitute too short time records to provide significant periods longer than 5 years (Fig. 3a,d). Wavelet coherence analysis based on neutron and tritium data for Kaitoke and Cuxhaven provides stronger evidence for an 11-year periodicity. In Fig. 4, all wavelet coherence analyses show an area of high spectral power in the middle of the time range. The period is also determined to be about 11 years.

### Artificial and natural effects for tritium in precipitation

These mathematical analyses provide the first evidence for the existence of 11-years periodicity of tritium and its correlation with secondary neutron flux for the period studied. Moreover, it is also important to consider other factors which might influence the magnitude of the temporal and spatial variation of the global tritium in the water cycle: 1. artificial effects: contribution of the bomb peak, and emissions from nuclear facilities; 2. natural effects: moisture source distribution, latitude effect of neutron flux, cloud formation, troposphere/stratosphere interactions, and contribution of surface-water evaporation to precipitation. We believe that artificial tritium emissions due to either weapon tests or nuclear facilities do not account for the pattern which we have shown to be synchronous with the solar cycle. Our previous study has demonstrated that an emission of 3.5 × 10^12^ Bq year^−1^ (1.8 × 10^21^ tritium atoms year^−1^) cannot be observed in precipitation farther than 5 km apart the source^38^.

Although the longest time series of Ottawa as well as precipitation in Hong Kong is suspected to have local contamination due to CANDU reactors^39^ (Supplementary Figs 1 and 2), it seems none of the tritium time series in our paper are affected by local tritium contamination from nuclear power plants, or by radioactive waste and reprocessing plants. In fact, Kaitoke is known to be free of these sources. Even precipitation at coastal locations at the North Sea, like Cuxhaven, are not significantly affected by local or regional contamination sources^40^. The attenuation of the bomb peak is still visible in the 1980’s, however the decreasing trend is similar in each sites, being devoid of oscillations attributed to local contaminations. The Cuxhaven precipitation is slightly affected by the moisture evaporated from North Sea, which is contaminated by artificial tritium from the Le Hague reprocessing plant^40^. However, this local contamination is not co-varying with the solar cycle, rather this gives a constant tritium offset to the meteoric precipitation at the coastal region of northern France, Belgium, The Netherlands and Germany.

However, natural processes might contribute to the tritium variation which have similar signature to an 11-year periodicity. Since tritium concentration depends on the geographical location of the precipitation as well as the moisture source, one has to consider whether long-term changes in the distribution of air mass sources may be linked to solar cycles. We can show that the distribution of moisture source carrying the tritium are independent of the solar magnetic variations, and these sources did not change in the last 25 years (See Supplementary).

To evaluate how solar cycles might affect the origin of the moisture sources, 120-hour backward trajectories have been calculated for our four locations (Supplementary Information)^41^. Altogether more than 14,000 trajectories (3,100 for Debrecen, 2,900 for Râmnicu Vâlcea, 4,800 for Cuxhaven and 3,200 for Wellington/Kaitoke) have been calculated using the HYSPLIT modelling system, representing all precipitation events between 1990 and 2014. For each year, the trajectories have been selected based on their source region and a frequency value has been calculated for all moisture source areas. Supplementary Fig. 5 shows how these frequencies vary with time for the four stations analysed in this study. For the European stations, five different source regions, while for Wellington three source regions could be defined. No long-term changes are present in the plots (Supplementary Fig. 5), indicating that none of the moisture source distributions have changed over the last 25 years. Hence it suggests that changes in the prevailing moisture source distribution have not influenced the visible long-term fluctuation of tritium concentration in precipitation. Consequently, shifts in the main moisture sources can be excluded as a main process driving tritium variability in precipitation.

Previous studies have shown that certain atmospheric processes, such as cloud and aerosol formation, stratosphere/troposphere coupling, are affected by solar variations^42–45^. Although it is very hard to assess the influence of these processes on the tritium concentrations in precipitation, we have made an attempt to evaluate positive feedback controlling the tritium content of precipitation.

## Discussion

**Figure 5:**
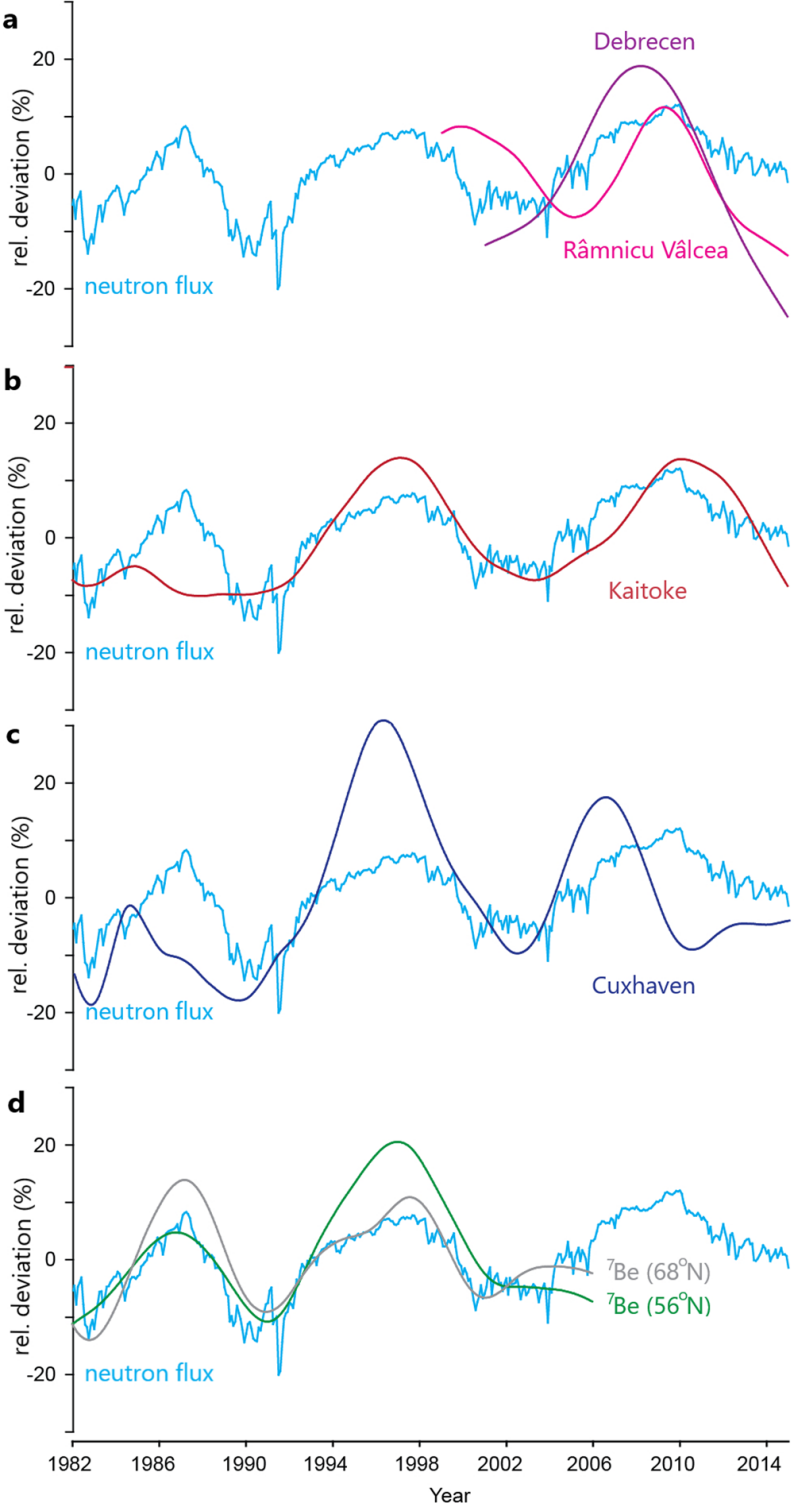
Temporal variations of neutron count rate (Oulu, Finland), tritium concentrations of precipitation and ^7^Be concentrations of atmospheric air. The values are expressed as a relative deviation compared to the long term averages. Note that the Kaitoke and Cuxhaven time series have been corrected for the contribution of the bomb peak. It can be obviously seen that neutron count rate and ^7^Be have an amplitude of 8–15% of the average, ^3^H might vary in a wider scale.

In order to further explore the natural variation of tritium in the precipitation and its relationship with other cosmogenic isotopes, the signal resulting from the nuclear weapon tests has been subtracted from the observed tritium concentration in Kaitoke and Cuxhaven. Figure 5 shows the deviations of the neutron flux, tritium and ^7^Be in different stations. While neutron monitor data are changing ±8–10% over the average, and ^7^Be is showing a very similar, but slightly wider trend, tritium varies over a wider range up to ±30%. Similar behaviour can be seen for ^10^Be. Neutron monitor data even at polar regions are sensitive to much higher energy of primary cosmic radiation (with an effective energy of 11–12 GeV) than beryllium isotopes (effective energy: 5.5–6.0 GeV)^46^. Therefore, it is expected that the magnitude of the 11-yr cycle in ^10^Be is greater than that in NM data. As for tritium variation, the magnitude is also higher than that of NM time series. This behaviour can be also attributed to the fact that production rate of cosmogenic tritium are sensitive to secondary neutrons of a different energy distribution (above 4 GeV).

However, the response of tritium variation in precipitation to the solar cycle also can help us understand what processes play a role in the water cycle from the tritium formation in the troposphere and stratosphere down to the surface^47,48^. Tritium is an important environmental tracer for understanding the global water cycle, and the tritium variation in precipitation that we found has implication for the use of cosmogenic tritium for dating young groundwater, e.g. understanding water flow through river and stream catchments. Nevertheless, further analysis needs to be performed over the next decades, in order to reveal the behaviour of tritium in precipitation related to solar effects. To study the correlation between solar cycle and tritium in precipitation, further precipitation as well as arctic or glacier ice cores, as an archive of precipitation accumulated before the nuclear era should also be investigated in the near future^49,50^. In this latter case very sensitive analytical capabilities with a precision of 0.005 TU or better^33,36,51^ will be required to overcome the analytical challenges given by the expected very low tritium amounts (0.1–0.2 TU) due to radioactive decay over the last >65 years.

## Methods

### Analytical methods

^3^H activities are expressed in tritium units (TU) where 1 TU represents a ^3^H/^1^H ratio of 1 × 10^−18^. In case of water, 1 TU equals to 0.119 Bq/dm^3^ activity concentration.

Cuxhaven and Debrecen precipitation samples have been analysed using the ^3^He ingrowth method^33,36^. This approach is based on the mass spectrometric measurement of the accumulation of ^3^He, the daughter product of tritium, from the decay of tritium. When analysing the Debrecen samples, a special isotope dilution technique was applied, where an ultrapure ^4^He spike was added to the sample in order to eliminate systematic errors^36,51^. The uncertainty of the ^3^He ingrowth method is about 0.1–0.4 TU for precipitation samples between 5–20 TU.

Râmnicu Vâlcea and Kaitoke samples have been analysed with liquid scintillation counting^34,35^. The Râmnicu Vâlcea samples have been measured with a Quantulus 1220 liquid scintillation counter. Due to the lack of tritium electrolytic enrichment for Râmnicu Vâlcea location, duplicate analyses were performed. It was decided to collect each individual precipitation in a month. The quantity corresponding to the collecting interval was recorded, and stored at low temperatures and at the end of the month both the individual precipitation for tritium concentration determination and the weighted mean of monthly precipitation were prepared, and they were measured in the same conditions. The tritium concentrations average of individual precipitation was calculated for current month and it was compared with the tritium concentration determined in the monthly composite precipitation. The reported values are the same (taking into account the measurement uncertainty) for both evaluations. This double-check allowed the use of recorded results even for results near the limit of detection.

Due to the use of electrolytic enrichment of the water, the Kaitoke samples (1–5 TU) have a better uncertainty of 0.03 TU. Samples for ^3^H were vacuum distilled and enriched by electrolysis prior to being analysed by liquid scintillation spectrometry using Quantulus ultra-low-level counters at GNS, New Zealand. Following the improvements from Morgenstern and Taylor (2009) the sensitivity is now further increased to a lower detection limit of 0.02 TU via tritium enrichment by a factor of 95, and reproducibility of tritium enrichment of 1% is achieved via deuterium-calibration for every sample^34^. The precision (1 sigma) is ~1.8% at 2 TU.

### Statistical methods

Pearson linear cross-correlation^52^ was calculated according to Equation 1 with Past 3.2 program available at https://folk.uio.no/ohammer/past/. For two time series x and y, the cross-correlation value at lag time m is:

1 $${{\rm{r}}}_{{\rm{m}}}=\frac{{\sum }^{}({{\rm{x}}}_{{\rm{i}}}-\bar{{\rm{x}}})({{\rm{x}}}_{{\rm{i}}-{\rm{m}}}-\bar{{\rm{y}}})\,}{\sqrt{{\sum }^{}{({{\rm{x}}}_{{\rm{i}}}-\bar{{\rm{x}}})}^{2}{\sum }^{}{({{\rm{y}}}_{{\rm{i}}-{\rm{m}}}-\bar{{\rm{y}}})}^{2}\,\,}\,}$$

The summations and the mean values are only taken over the parts where the sequences overlap for a given time lag. The equation shows that for positive lags, x is compared with a y that has been delayed by m samples. A high correlation value at positive lags thus means that features in y are leading, while x lags behind. For negative lags, features in x are leading. Cross-correlation significance has been calculated by the Ebisuzaki method^53^. The Ebisuzaki open source code is available at ftp://ftp.cpc.ncep.noaa.gov/wd51we/random_phase/. The spline smoothing was done using Past 3.2 program with bomb-peak correction used as follows: In the first step, we approximated an attenuating trend of the bomb tritium with an exponential function. In the second step, we calculated the bomb-peak corrected (BPC) time series as the subtraction of the tritium time series and the exponential function. The parameters of this exponential function have been determined with an optimisation algorithm.

The application of wavelet transformation has became widespread in the non-linear time series analysis. This method was applied in our time series. We used the Mexican Hat wavelet function (second derivative of Gaussian; DOG m = 2) because this function is covered better by the time series (Fig. 4.) than the Morlet function (derived from the Gaussian function). In addition, the Mexican Hat provides better detection and localization of patch and gap events over the Morlet function. Peaks and troughs in the scalogram of WAC (relatively extreme values of the wavelet coefficient) from Mexican hat can be directly linked to events and site conditions. In contrast, the Morlet offers improved scale detection and localization over the Mexican Hat. The open source MATLAB code of Grinsted *et al*. (2004) was applied to produce the figures^37^.

## Electronic supplementary material

Supplementary Information

Dataset 1
