# Supplementary material for: Modulation of Cosmogenic Tritium in Meteoric Precipitation by the 11-year Cycle of Solar Magnetic Field Activity
Source: Sci Rep. 2018 Aug 24;8:12813. doi: 10.1038/s41598-018-31208-9 (PMC6109153; doi:10.1038/s41598-018-31208-9)
Supplement: Supplementary file 1 — Supplementary Information [file 41598_2018_31208_MOESM1_ESM.docx]

**Modulation of Cosmogenic Tritium in Meteoric Precipitation by the 11-year Cycle of Solar Magnetic Field Activity**

**László Palcsu^1,*^, Uwe Morgenstern^2^, Jürgen Sültenfuss^3^, Gabriella Koltai^1,4^, Elemér László^1^, Marjan Temovski^1^, Zoltán Major^1^, Judit T. Nagy^5^, László Papp^1^, Carmen Varlam^6^, Ionut Faurescu^6^, Marianna Túri^1^, László Rinyu^1^, György Czuppon^7^, Emese Bottyán^7^, A.J. Timothy Jull^1,8^**

^1^Isotope Climatology and Environmental Research Centre (ICER), Institute for Nuclear Research, Hungarian Academy of Sciences, Debrecen, 4026 Hungary

^2^GNS Science, Lower Hutt, 5040 New Zealand

^3^Institute of Environmental Physics, University of Bremen, Bremen, 28359 Germany

^4^Institute of Geology, University of Innsbruck, Innsbruck, 6020 Austria

^5^Doctoral School of Mathematical and Computational Sciences, University of Debrecen, Debrecen, 4032 Hungary

^6^National R&D Institute of Cryogenics and Isotopic Technologies, Râmnicu Vâlcea, 240050 Romania

^7^Institute for Geological and Geochemical Research, Research Centre for Astronomy and Earth Sciences, Hungarian Academy of Sciences, Budapest, 1112 Hungary

^8^Department of Geosciences, University of Arizona, Tucson, AZ 85721 USA

*palcsu.laszlo@atomki.mta.hu

**The tritium bomb-peak**


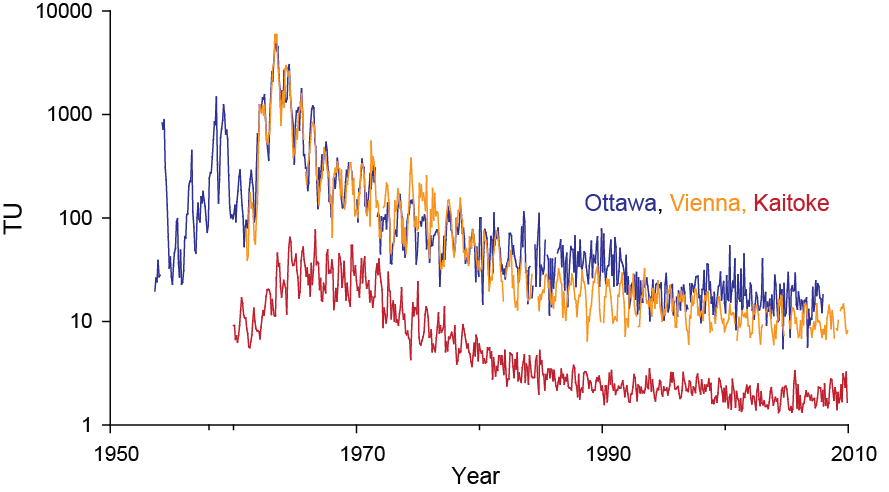


Figure 1. The longest tritium time series from the northern hemisphere: Ottawa (Canada) and Vienna (Austria), and the southern hemisphere: Kaitoke (New Zealand). Ottawa samples might be contaminated by local tritium emissions of CANDU reactors, while the Vienna and Kaitoke time series are free of contamination.

**Frequency distribution and cross correlation**


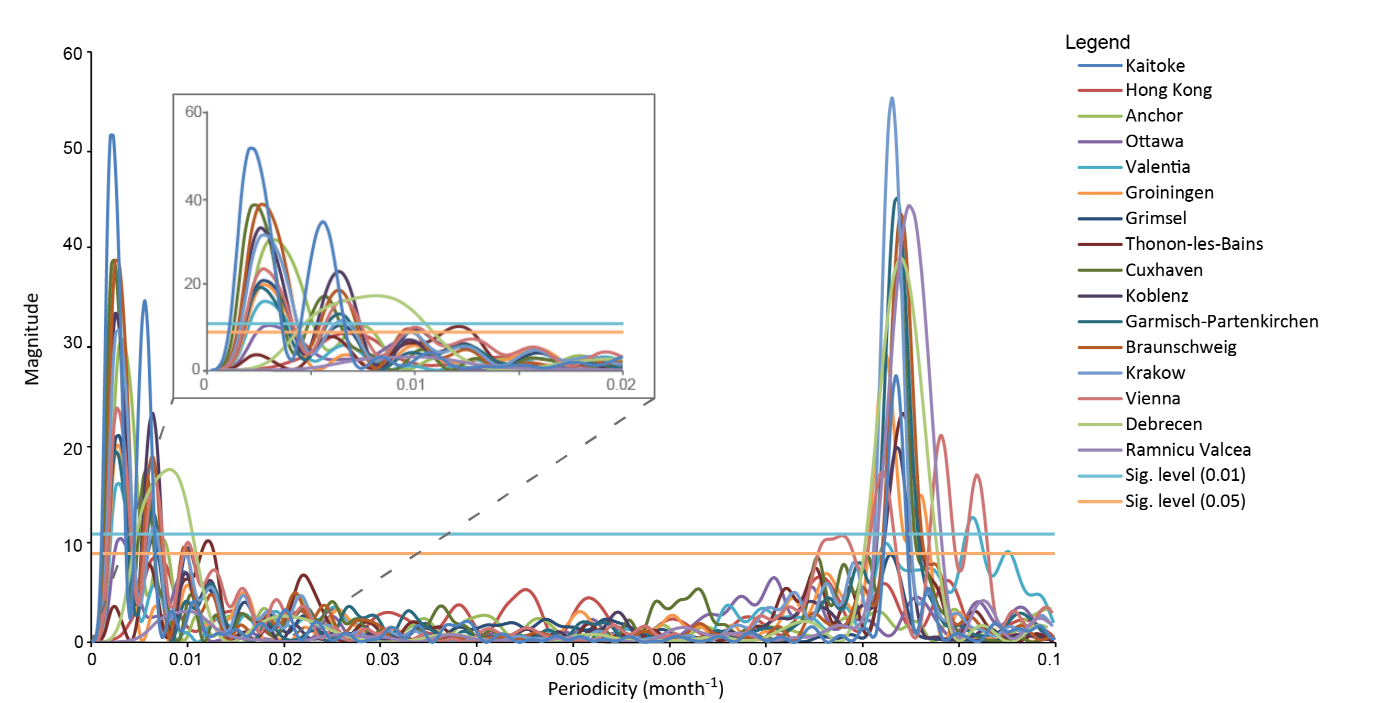


Figure 2. Frequency (periodicity) analysis of the tritium data (including Ottawa and Hong Kong) from 1980 to 2014. An apparent frequency appears at 0.0025 month^-1^ (~33 years of periodicity), which is attributed to the attenuation of the bomb peak.

**Cross correlation**

**
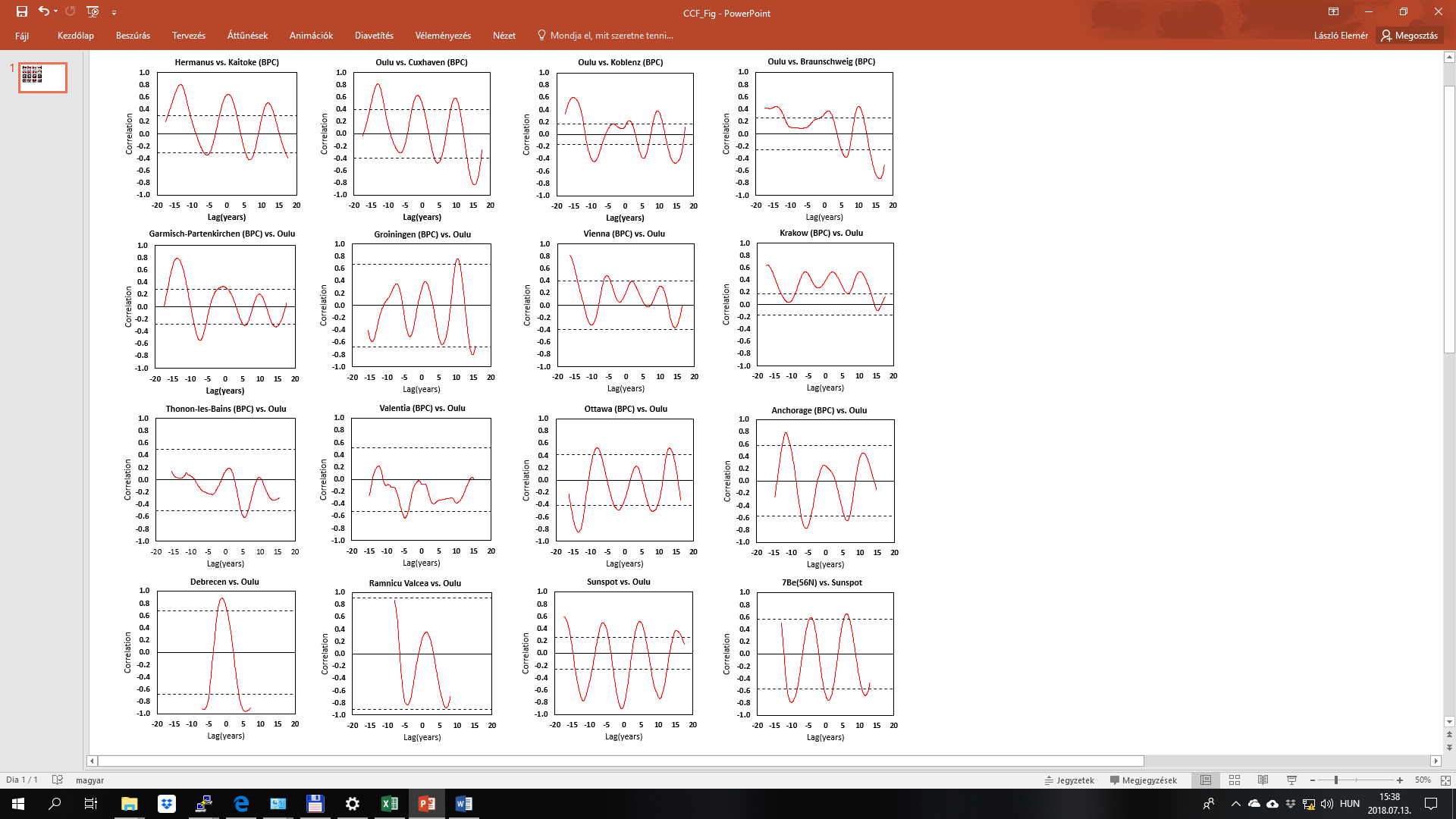
**

Figure 3. Cross-correlation function (CCF) between neutron count rate, tritium concentration of precipitation and Sunspot number (dashed lines denote the 95% confidence interval): Note that all CCF have been made with bomb peak correction (BPC).

**Description of the trajectory calculation**

For Debrecen, the HYSPLIT model (HYbrid Single-Particle Lagrangian Integrated Trajectory developed by the National Oceanic and Atmospheric Administration Air Resources Laboratory; Draxler and Rolph, 2003) was applied to produce the 96-hour backward trajectories for all rainy events between 2001 and 2014 using the ERA-Interim database (Dee et al., 2011) with appr. 80 km spatial resolution on 60 vertical levels.

The backward trajectories were calculated for every day and every hour when precipitation was observed for heights of 500, 1500 and 3000 m. In order to evaluate the right time interval of the precipitation event a threshold for the relative humidity was applied. Only those trajectories were considered for further calculations, where the relative humidity reached 80% at the starting point (Sodemann et al., 2008b).

To determine the moisture uptake region, the Lagrangian moisture source diagnostic was carried out by calculating the specific humidity along the backward trajectories. When the specific humidity increased within a time interval, the overpassed area was considered as moisture source location. Summarising up these source locations, we could estimate the distribution of the different source regions (Tropical, Atlantic, Mediterranean, Black Sea, Northern Seas, Pannon Basin and other continental areas, Suppl. Fig. 5). To produce the trajectories for other locations, also the HYSPLIT model was applied. In these cases the backward trajectories have been calculated using the online version of HYSPLIT v4.0 using the database provided by the Global Data Assimilation System (GDAS) with 1° spatial resolution. More details are available at: https://www.ncdc.noaa.gov/data-access/model-data/model-datasets/global-data-assimilation-system-gdas.

**
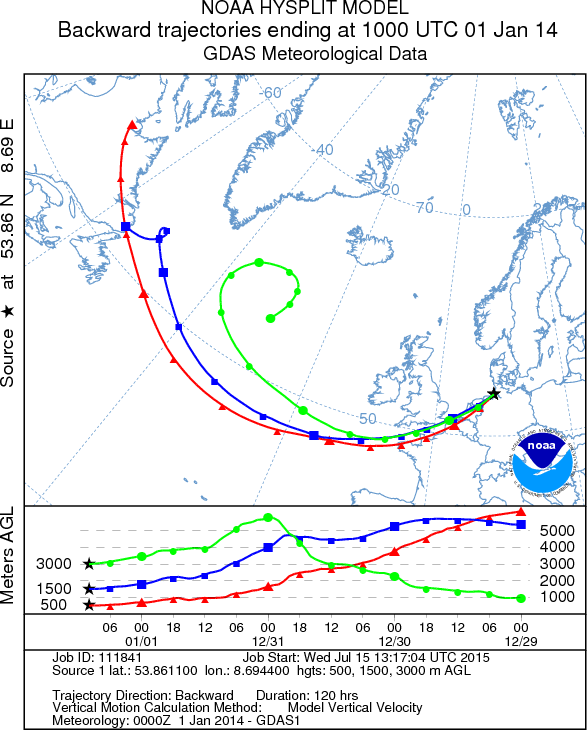
**

Figure 4. Example of a HYSPLIT backward trajectory in case Cuxhaven.

Draxler, R. R. and Hess, G. D., 1997: Description of the Hysplit_4 modeling system. NOAA Tech Memo ERL ARL-224, 1997 Dec, 24 p.

Draxler, R.R. and Rolph, G.D., 2003. HYSPLIT (HYbrid Single-Particle Lagrangian Integrated Trajectory) Model access via NOAA ARL READY Website (http://www.arl.noaa.gov/ready/hysplit4.html). NOAA Air Resources Laboratory, Silver Spring, MD.

Dee, D.P., Uppala, S.M., Simmons, A.J., Berrisford, P., Poli, P., Kobayashi, S., Andrae, U., Balmaseda, M.A., Balsamo, G., Bauer, D.P. and Bechtold, P., 2011. The ERA‐Interim reanalysis: Configuration and performance of the data assimilation system. *Quarterly Journal of the royal meteorological society*, *137*(656), pp.553-597.

Sodemann, H., Schwierz, C. and Wernli, H., 2008. Interannual variability of Greenland winter precipitation sources: Lagrangian moisture diagnostic and North Atlantic Oscillation influence. *Journal of Geophysical Research: Atmospheres*, *113*(D3).

**Distribution of moisture source regions**


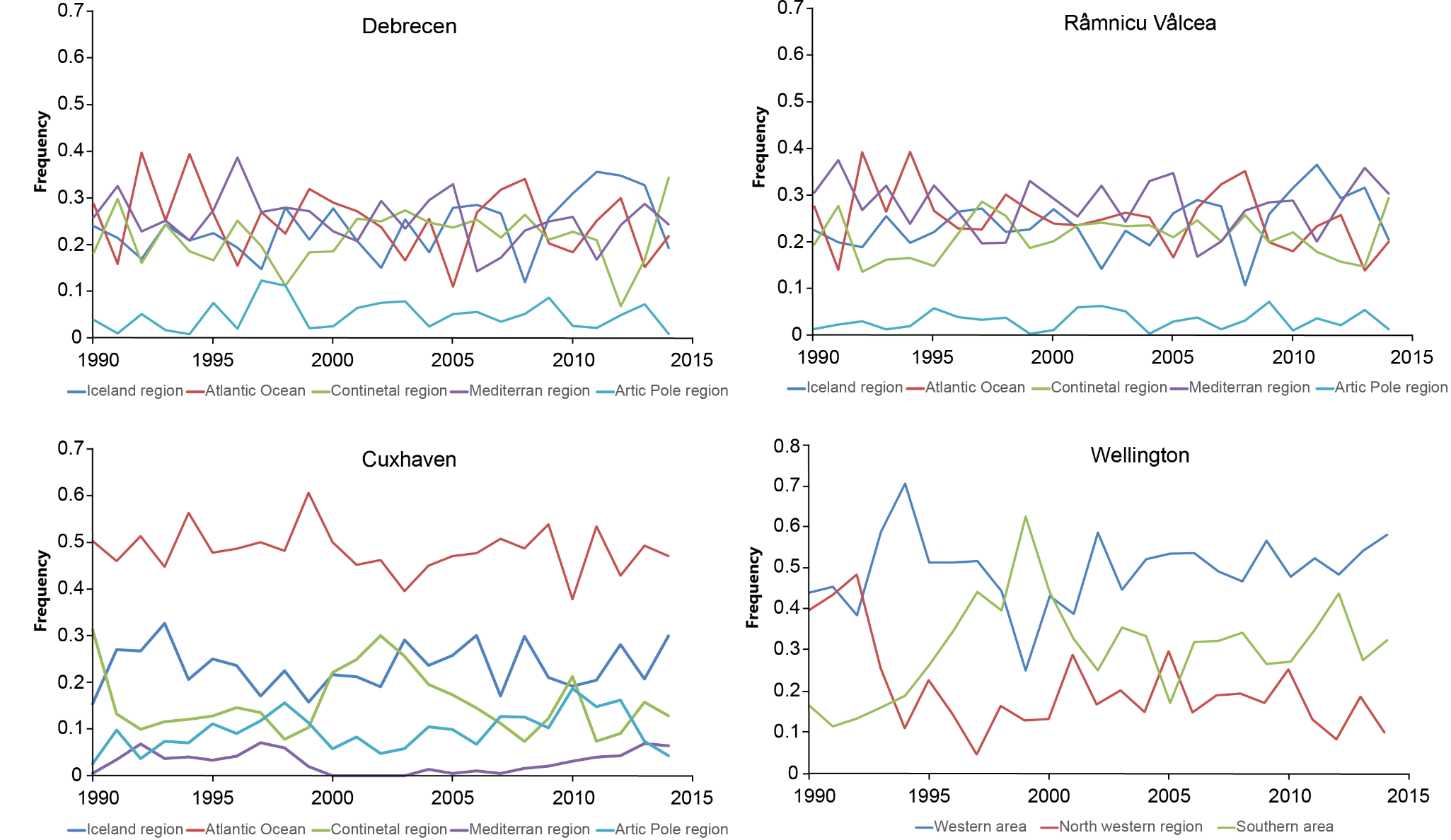


Figure 5. Frequencies of the moisture source regions for four locations between 1990 and 2014. No significant change can be seen at any stations that could be attributed to solar cycle.
